# Estimation of local time-varying reproduction numbers in noisy surveillance data

**DOI:** 10.1101/2021.04.23.21255958

**Published:** 2021-04-27

**Authors:** Wenrui Li, Katia Bulekova, Brian Gregor, Laura F. White, Eric D. Kolaczyk

**Affiliations:** 1 Department of Mathematics and Statistics, Boston University, Boston MA, USA; 2 Research Computing Services, Information Services and Technology, Boston University, Boston MA, USA; 3 Department of Biostatistics, Boston University, Boston MA, USA; 4 Hariri Institute for Computing, Boston University, Boston MA, USA

## Abstract

A valuable metric in understanding infectious disease local dynamics is the local time-varying reproduction number, i.e. the expected number of secondary local cases caused by each infected individual. Accurate estimation of this quantity requires distinguishing cases arising from local transmission from those imported from elsewhere. Realistically, we can expect identification of cases as local or imported to be imperfect. We study the propagation of such errors in estimation of the local time-varying reproduction number. In addition, we propose a Bayesian framework for estimation of the true local time-varying reproduction number when identification errors exist. And we illustrate the practical performance of our estimator through simulation studies and with outbreaks of COVID-19 in Hong Kong and Victoria, Australia.

## Introduction

Epidemic modeling, while not at all new, has taken on renewed importance due to the COVID-19 pandemic. The local time-varying reproduction number, $R_{*}^{\text{local }}(t)$, is an important quantity to monitor the infectiousness and transmissibility of diseases and, therefore, to design and adjust public health responses during an outbreak. Recent examples include monitoring transmission of the COVID-19 pandemic and demonstrating the efficacy of non-pharmaceutical interventions in more than 100 countries [1–4]. The value of $R_{*}^{\text{local }}(t)$ represents the expected number of secondary local cases arising from a primary case infected at time *t*. Different formal definitions of $R_{*}^{\text{local }}(t)$ have been proposed, and a number of methods are available to estimate this quantity. The most widely used is an estimator of the instantaneous reproduction number that is defined as the ratio of the expected number of incident locally infected cases at time *t* to the expected total infectiousness of infected individuals at time *t* [5, 6].

Distinguishing local cases from imported cases is essential to estimation of the local time-varying reproduction number. However, surveillance data generally is available only up to some level of error. For example, if we are unable to identify the correct source of infection from contact tracing or genetic information, imported cases might be misclassified as local cases, and vice versa. Such misclassification error is recognized as one limitation of estimating $R_{*}^{\text{local }}(t)$ in the COVID-19 outbreak [7, 8]. We investigate how identification error impacts on the estimation of the instantaneous reproduction number and, thus, on our understanding of diseases transmission dynamics.

Extensive work regarding improving inference of time-varying reproduction numbers has been done. For instance, there have been efforts to estimate the serial interval that is used to compute the total infectiousness for $R_{*}^{\text{local }}(t)$ estimation, including Bayesian parametric estimation using data augmentation Markov Chain Monte Carlo [9], and a cure model for limited follow-up data [10]. Many studies have explored the effects of imperfect detection and estimated the true infection prevalence [8, 11–13]. But, to our best knowledge, there has been little attention to date given towards accounting for identification errors of local and imported cases.

Our contribution in this paper is to quantify how such errors propagate to the local time-varying reproduction number, and to provide estimators for $R_{*}^{\text{local }}(t)$ when contact tracing survey information is available. Adopting the definition of $R_{*}^{\text{local }}(t)$ proposed by [5], we characterize the impact of identification errors on the bias of noisy local time-varying reproduction numbers. Our work shows that, in general, the bias can be expected to be nontrivial. Accordingly, we propose a Bayesian framework to estimate the true local time-varying reproduction number. Numerical simulation suggests that high accuracy is possible for estimating local time-varying reproduction numbers in outbreaks of even modest size. We illustrate the practical use of our estimators in the context of COVID-19 pandemic in Hong Kong and Victoria, Australia.

The organization of this paper is as follows. In Methods Section we show the bias of the noisy local time-varying reproduction number, and propose a Bayesian hierarchical framework to estimate the true local time-varying reproduction number with imperfect knowledge. Results Section reports the practical performance of our estimators through simulation studies and with SARS-CoV-2 infections in Hong Kong and Australia. Finally, we conclude in Discussion Section with a discussion of future directions for this work.

## Methods

In this section, we first quantify the bias of the noisy local time-varying reproduction number when misidentification occurs in the surveillance data. We then build a Bayesian hierarchical framework to estimate true local time-varying reproduction numbers. We also propose a method to estimate misidentification rates based on contact tracing survey data, which informs the prior distribution in the model.

### Notation

We provide essential notation and background here. The number of newly infected cases at time *t*, I_*_(*t*), is the sum of the numbers of local $\left(I_{*}^{\text{local }}(t)\right)$ and imported $\left(I_{*}^{\text{imported }}(t)\right)$ cases. If one assumes independence between calendar time and the generation interval, *g*(*s*), then the local time-varying reproduction number is defined as [5]

(1) $$R_{*}^{\text{local }}(t)\;=\;\frac{\mu_{*}^{\text{local }}(t)}{\int_{0}^{\infty }g(s)\mu_{*}(t\;-\;s)ds},$$

where $\mu_{*}^{\text{local }}(t)\;=\;E\left[I_{*}^{\text{local }}(t)\right]$ and $\mu_{*}(t)\;=\;E\left[I_{*}(t)\right]$.

In reality, we only know the serial interval and the number of diagnosed cases. Let *I*(*t*), *I*^local^(*t*) and *I*^imported^(*t*) be the numbers of total diagnosed cases, local diagnosed cases, and imported diagnosed cases at time *t*, respectively. Then, we define a realistic local time-varying reproduction number as

(2) $$R^{\text{local }}(t)\;=\;\frac{\mu^{\text{local }}(t)}{\int_{0}^{\infty }w(s)\mu (t\;-\;s)ds},$$

where *w*(*s*) is the serial interval, $\mu^{\text{local }}(t)\;=\;E\left[I^{\text{local }}(t)\right]$ and μ(t)=E[I(t)]. Note that the serial interval corresponds to date of symptom onset. One can estimate symptom onset dates by back calculation of report dates [14].

Realistically, we can expect identification of cases as local or imported to be imperfect. Let *Ĩ*^local^(*t*) and *Ĩ*^imported^(*t*) be the number of new local and imported cases reported at time *t*, with identification error. Thus, we define a noisy local time-varying reproduction number as

(3) $${\tilde{R}}^{\text{local }}(t)\;=\;\frac{{\tilde{\mu }}^{\text{local }}(t)}{\int_{0}^{\infty }w(s)\mu (t\;-\;s)ds},$$

where ${\tilde{\mu }}^{\text{local }}(t)\;=\;E\left[{\tilde{I}}^{\text{local }}(t)\right]$. The definition of ${\tilde{R}}^{\text{local }}(t)$ in (3) comes from an argument that mimics the original argument using Poisson arrivals in [15]. Specifically, we suppose that we observe a Poisson stream ${\tilde{I}}^{\text{local }}(t)$ that is a function of calendar time *t* in terms of the transmissibility, denoted ${\tilde{\beta }}^{\text{local }}(t,\;s)$, an arbitrary function of calendar time *t* and time since infection *s*. Then, ${\tilde{\mu }}^{\text{local }}(t)$ follows the so-called renewal equation

(4) $${\tilde{\mu }}^{\text{local }}(t)\;=\;\int_{0}^{\infty }{\tilde{\beta }}^{\text{local }}(t,\;s)\mu (t\;-\;s)ds.$$

Following [15], we have

(5) $${\tilde{\beta }}^{\text{local }}(t,\;s)\;=\;{\tilde{R}}^{\text{local }}(t)w(s).$$

Inserting (5) into (4) yields the definition of ${\tilde{R}}^{\text{local }}(t)$ in (3).

Our interest is in characterizing the manner in which the uncertainty in *Ĩ*^local^(*t*) and *Ĩ*^imported^(*t*) propagates to the local time-varying reproduction number, and providing estimators of *R*^local^(*t*) to account for identification errors.

### Bias of the noisy local time-varying reproduction number

We quantify the bias of the noisy local time-varying reproduction number in (3) when misidentification occurs. We begin by defining a model for *Ĩ*^local^(*t*) and *Ĩ*^imported^(*t*). Let *α*_0_ denote the probability that an imported case is misidentified as local, and *α*_1_ the probability that a local case is misidentified as imported. Then, a simple model is

(6) $${\tilde{I}}^{\text{local }}(t)|I^{\text{local }}(t),\;I^{\text{imported }}(t),\;\alpha_{0},\;\alpha_{1}\;\sim \;\text{Bin}\left(I^{\text{local }}(t),\;1\;-\;\alpha_{1}\right)\;+\;\text{Bin}\left(I^{\text{imported }}(t),\;\alpha_{0}\right),\;{\tilde{I}}^{\text{imported }}(t)\;=\;I^{\text{local }}(t)\;+\;I^{\text{imported }}(t)\;-\;{\tilde{I}}^{\text{local }}(t).$$

Under independence, the first relationship in (6) is directly obtained by the definition of *α*_0_ and *α*_1_. And the second equation in (6) is due to the fact that the total number of cases reported at time *t* is not affected by the misidentification.

By (6), the relationship between ${\tilde{\mu }}^{\text{local }}(t)$ and *μ*^local^(*t*) is

(7) $${\tilde{\mu }}^{\text{local }}(t)\;=\;\left(1\;-\;\alpha_{1}\right)\mu^{\text{local }}(t)\;+\;\alpha_{0}\mu^{\text{imported }}(t),$$

where $\mu^{\text{imported }}(t)\;=\;E\left(I^{\text{imported }}(t)\right)$. Direct computation yields

(8) $${\tilde{R}}^{\text{local }}(t)\;=\;\left(1\;-\;\alpha_{1}\;+\;\alpha_{0}\frac{\mu^{\text{imported }}(t)}{\mu^{\text{local }(t)}}\right)R^{\text{local }}(t)$$

when *μ*^local^(*t*) ≠ 0. From (8), we can see that the bias of ${\tilde{R}}^{\text{local }}(t)$ depends on *α*_0_, *α*_1_ and the ratio of *μ*^imported^(*t*) and *μ*^local^(*t*). When *μ*^imported^(*t*)/*μ*^local^(*t*) = 1, we have ${\tilde{R}}^{\text{local}}(t)\;>\;R^{\text{local}}(t)$ if *α*_0_ > *α*_1_, and ${\tilde{R}}^{\text{local}}(t)\;<\;R^{\text{local}}(t)$ if *α*_0_ < *α*_1_.

### Bayesian hierarchical modeling to account for misidentification

We propose a Bayesian framework to estimate *R*^local^(*t*) using noisy surveillance data. Following [5, 6, 15], we specify

(9) $$I^{\text{local}}(t)|R^{\text{local }}(t),\;n(t\;-\;1),\;w(s)\;\sim \;\text{Pois}\left(R^{\text{local }}(t)\;\cdot \;\Lambda (t)\right),\;\text{for}\;t\;>\;0,$$

where $\Lambda (t)\;=\;\sum_{s=1}^{t}w(s)I(t\;-\;s)$ is the total infectiousness of infected individuals at time *t*, and *n*(*t*−1) represent the historical data up to time *t*−1 (i.e., *I*^local^(0), *I*^imported^(0), · · ·, *I*^local^(*t* − 1), *I*^imported^(*t* − 1)). Note that Λ(*t*) is undefined for *t* = 0. So, we assume that

(10) $$I^{\text{local}}\;(0)|\mu^{\text{local }}(0)\;\sim \;\text{Pois}\left(\mu^{\text{local }}(0)\right).$$

And we assume the imported case counts follow a Poisson distribution:

(11) $$I^{\text{imported }}(t)|\mu^{\text{imported }}(t)\;\sim \;\text{Pois}\left(\mu^{\text{imported }}(t)\right).$$

Next, we define relevant prior distributions. We assume a distribution for *R*^local^(*t*) of the form

(12) $$R^{\text{local }}(t)|n(t\;-\;1),\;w(s)\;\sim \;\text{Gamma}\left(a_{t|t-1}^{\text{local }},\;b_{t|t-1}^{\text{local }}\right),\;\text{for}t\;>\;0.$$

This choice is similar to that in [5], but differs in that we specify gamma conditioned on the history, rather than marginally. The conditioning reflects the expectation that the evolution of *R*^local^(*t*) is likely to depend on the course of infection in the population and intervention measures that may result. Analogously, we also assume gamma distributed priors for *μ*^imported^(*t*) and *μ*^local^(0), that is,

(13) $$\mu^{\text{imported }}(t)\;\sim \;\text{Gamma}\left(a_{t}^{\text{imported }},\;b_{t}^{\text{imported }}\right),\;\mu^{\text{local }}(0)\;\sim \;\text{Gamma}\left(a_{0}^{\text{local }},\;b_{0}^{\text{local }}\right).$$

In addition, we assume the convention that the misidentification rates are beta distributed, and hence given by

(14) $$\alpha_{0}\;\sim \;\text{Beta}\left(\zeta_{\alpha_{0}},\;\xi_{\alpha_{0}}\right),\;\alpha_{1}\;\sim \;\text{Beta}\left(\zeta_{\alpha_{1}},\;\xi_{\alpha_{1}}\right).$$

By using Markov chain Monte Carlo (MCMC) simulation, we can get both estimates of *R*^local^(*t*) and its uncertainty. We implement MCMC using the R package, NIMBLE [16–18] with the default assignment of sampler algorithms. The samplers assigned to the variables are as follows: Gibbs samplers are assigned to *μ*^local^(0) and *μ*^imported^(*t*), *t* ≥ 0, which have conjugate relationships between their prior distribution and the distributions of their stochastic dependents; slice samplers [19] are used for *I*^local^(*t*) and *I*^imported^(*t*), *t* ≥ 0; Metropolis-Hastings adaptive random-walk samplers are set to *α*_0_, *α*_1_ and *R*^local^(*t*), *t* > 0.

### Estimating misidentification rates

Without any information on the misidentification rates, it is difficult to get an accurate estimator of *R*^local^(*t*). However, contact tracing data could provide adequate information to estimate the misidentification rates.

Let *p*_*i*_ be the probability that we think individual *i* is a local case based on the survey. Then, *p*_*i*_ can be modeled as a mixture of *α*_0_ and 1 − *α*_1_. Note that $\alpha_{1}\;\sim \;\text{Beta}\left(\zeta_{\alpha_{1}},\;\xi_{\alpha_{1}}\right)$ implies $1\;-\;\alpha_{1}\;\sim \;\text{Beta}\left(\xi_{\alpha_{1}},\;\zeta_{\alpha_{1}}\right)$. We thus model the distribution of *p*_*i*_ as a mixture of two beta distributions:

(15) $$p_{i}\;\sim \;\pi_{0}\text{Beta}\left(\zeta_{\alpha_{0}},\;\xi_{\alpha_{0}}\right)\;+\;\left(1\;-\;\pi_{0}\right)\text{Beta}\left(\xi_{\alpha_{1}},\;\zeta_{\alpha_{1}}\right),$$

where *π*_0_ can be interpreted as the fraction of the diagnosed cases that are imported. By using the expectation–maximization (EM) algorithm, we can obtain estimators ${\hat{\zeta }}_{\alpha_{0}}$, ${\hat{\xi }}_{\alpha_{0}}$, ${\hat{\zeta }}_{\alpha_{1}}$ and ${\hat{\xi }}_{\alpha_{1}}$.

Note that, if $1\;-\;\zeta_{\alpha_{0}}/\left(\zeta_{\alpha_{0}}\;+\;\xi_{\alpha_{0}}\right)\;-\;\zeta_{\alpha_{1}}/\left(\zeta_{\alpha_{1}}\;+\;\xi_{\alpha_{1}}\right)\;\neq \;0$, we obtain unbiased estimators of *I*^local^(*t*) and *I*^imported^(*t*)

(16) $${\hat{I}}^{\text{local }}(t)\;=\;\frac{\left[1\;-\;\zeta_{\alpha_{0}}/\left(\zeta_{\alpha_{0}}\;+\;\xi_{\alpha_{0}}\right)\right]\;\cdot \;{\tilde{I}}^{\text{local }}(t)\;-\;\zeta_{\alpha_{0}}/\left(\zeta_{\alpha_{0}}\;+\;\xi_{\alpha_{0}}\right)\;\cdot \;{\tilde{I}}^{\text{imported }}(t)}{1\;-\;\zeta_{\alpha_{0}}/\left(\zeta_{\alpha_{0}}\;+\;\xi_{\alpha_{0}}\right)\;-\;\zeta_{\alpha_{1}}/\left(\zeta_{\alpha_{1}}\;+\;\xi_{\alpha_{1}}\right)},\;{\hat{I}}^{\text{imported }}(t)\;=\;\frac{\left[1\;-\;\zeta_{\alpha_{1}}/\left(\zeta_{\alpha_{1}}\;+\;\xi_{\alpha_{1}}\right)\right]{\tilde{I}}^{\text{imported }}(t)\;-\;\zeta_{\alpha_{1}}/\left(\zeta_{\alpha_{1}}\;+\;\xi_{\alpha_{1}}\right){\tilde{I}}^{\text{local }}(t)}{1\;-\;\zeta_{\alpha_{0}}/\left(\zeta_{\alpha_{0}}\;+\;\xi_{\alpha_{0}}\right)\;-\;\zeta_{\alpha_{1}}/\left(\zeta_{\alpha_{1}}\;+\;\xi_{\alpha_{1}}\right)}.$$

Thus, good initial values of *I*^local^(*t*) and *I*^imported^(*t*) in MCMC are estimators of *Î*^local^(*t*) and *Î*^imported^(*t*) based on the estimated misidentification rates, i.e., replacing $\zeta_{\alpha_{0}}$, $\xi_{\alpha_{0}}$, $\zeta_{\alpha_{1}}$, $\xi_{\alpha_{1}}$ in (16) by ${\hat{\zeta }}_{\alpha_{0}}$, ${\hat{\xi }}_{\alpha_{0}}$, ${\hat{\zeta }}_{\alpha_{1}}$, ${\hat{\xi }}_{\alpha_{1}}$.

## Results

In this section, we conduct some simulations to illustrate the performance of the proposed estimation methods. And we apply our method to two real data sets. One is surveillance data of COVID-19 in Hong Kong that includes contact tracing information, including travel history data [20]. They collected information on 1,038 SARS-CoV-2 cases confirmed between 23 January and 28 April 2020. And they identified 355 local cases and 683 imported cases. The other data set is from the COVID-19 pandemic in Victoria, Australia, studied in [21]. There they had 1,333 laboratory-confirmed cases of COVID-19 between 6 January and 14 April 2020. After excluding duplicate patients from cases, they identified 345 local cases and 558 imported cases.

We consider two settings, a simulation setting and an application setting. In the simulation setting, we first use surveillance data from Hong Kong and Victoria to create realistic simulated data, and then we add identification errors to the ‘true’ local and imported cases derived from the simulated epidemics, finally we estimate the local time-varying reproduction number using the noisy local and imported cases counts. In the application setting, we assume that identified local and imported cases in the real data sets are with some error. The former results allow us to understand what properties can be expected of our estimators, while the latter are reflective of what would be observed in practice with such data.

### Simulation study

**Fig 1.**
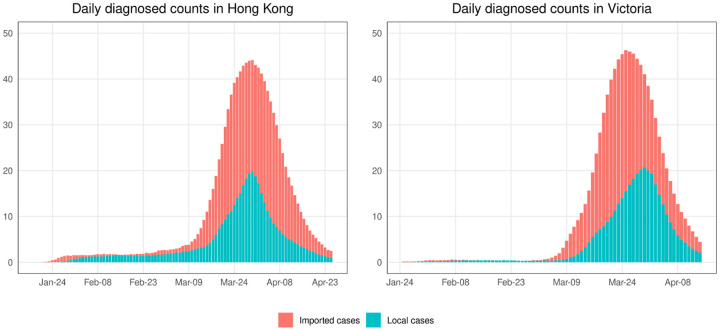
The means of daily local and imported diagnosed counts in 1,000 simulation trials for epidemics in Hong Kong and Victoria.

In this simulation study, we used Covasim [22], a stochastic individual-based model for transmission of SARS-CoV-2, calibrated to the epidemics in Hong Kong and Victoria. Fig 1 shows the average daily local and imported diagnosed counts over 1,000 trials. The noisy *Ĩ*^local^(*t*) and *Ĩ*^imported^(*t*) are generated according to (6). We set *α*_0_ ~ Beta(2, 18) (mean of 0.1), and *α*_1_ ~ Beta(2, 8) (mean of 0.2), Beta(4, 8) (mean of 0.33), or Beta(8, 8) (mean of 0.5) to see the effect of small *α*_0_ and large *α*_1_. This might happen if the definition of imported cases relies on travel history collected in the case investigation and some people are infected locally, even though they have a travel history within 14 days prior to symptom onset. We also consider *α*_1_ ~ Beta(2, 18), and *α*_0_ ~ Beta(2, 8), Beta(4, 8), or Beta(8, 8) (corresponding to small *α*_1_ and large *α*_0_, which might occur if cases are defined as local when we are not sure about their source of infection.) We assume that both *α*_0_ and *α*_1_ are unknown.

**Fig 2.**
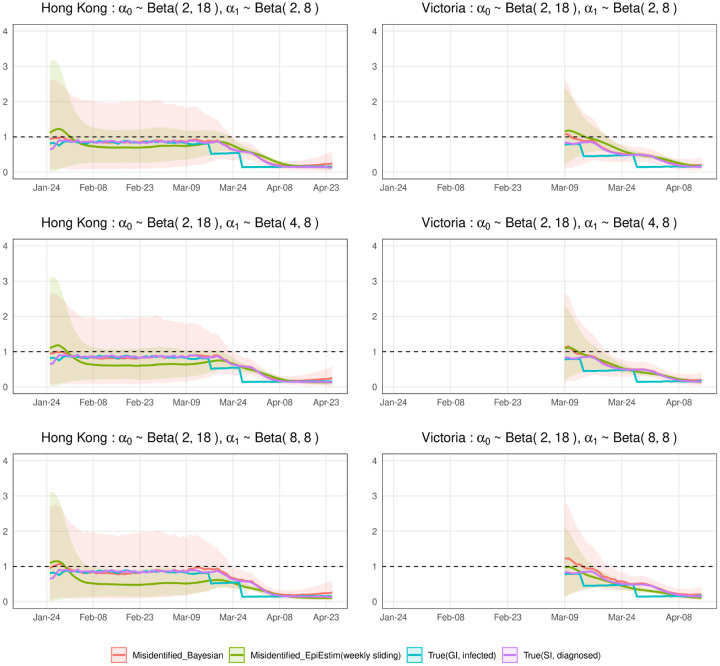
Estimations of local time-varying reproduction numbers in simulated epidemics for Hong Kong and Victoria under three sets of error misidentification rates: *α*_0_ ~ Beta(2, 18), and *α*_1_ ~ Beta(2, 8), Beta(4, 8), or Beta(8, 8). The error bands are the averages of 95% credible intervals over 1,000 trials. Note that the differences between the blue curve $\left(R_{*}^{\text{local }}(t)\right)$ and the purple curve (*R*^local^(*t*)) are due to the differences among infected dates, symptom onset dates, diagnosed dates.

**Fig 3.**
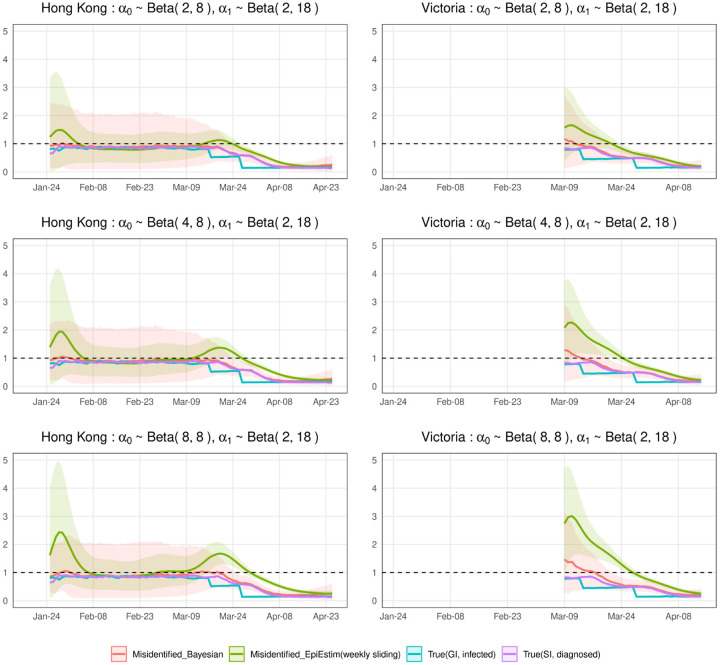
Estimations of local time-varying reproduction numbers in simulated epidemics for Hong Kong and Victoria under three sets of error misidentification rates: *α*_1_ ~ Beta(2, 18), and *α*_0_ ~ Beta(2, 8), Beta(4, 8), or Beta(8, 8). The error bands are the averages of 95% credible intervals over 1,000 trials.

We evaluate the estimate for *R*^local^(*t*) in terms of a corresponding posterior, and 95% credible intervals. Fig 2 and 3 show the simulation results, in which we run MCMC chains of 10,000 samples for each of 1,000 simulated epidemic trials. Fig 2 assumes that we are more likely to misclassify local cases as imported cases and Fig 3 assumes that we are more likely to misclassify imported cases as local cases. For comparison purposes, we compute ${\tilde{R}}^{\text{local }}(t)$ and *R*^local^(*t*) defined in (1) and (2) by approximating $\mu_{*}^{\text{local }}(t)$, μ_*_(*t*), *g*(*s*), *μ*^local^(*t*), *μ*(*t*), *w*(*s*) using 1,000 simulation trials. And we calculate the most widely used estimator of ${\tilde{R}}^{\text{local }}(t)$ defined in (3), which is implemented in the R package, EpiEstim [23]. We view it as a representative estimator that does not account for misidentification, i.e., it treats the noisy local and imported cases as true.

In the simulated epidemics for both Hong Kong and Victoria, if we ignore the misidentification, we will underestimate *R*^local^(*t*) when the mean of *α*_0_ is small and the mean of *α*_1_ is relatively large (Fig 2), and overestimate *R*^local^(*t*) when the mean of *α*_1_ is small and the mean of *α*_0_ is relatively large (Fig 3), with the biases increasing when the means of *α*_0_ and *α*_1_ increase. The results are consistent with (8) implying that the biases will lead to inappropriate public health response, i.e., inadequate interventions or overreaction. We correct the bias by our Bayesian hierarchical framework. The biases of our estimators are close to zero in all cases. The 95% credible intervals of our estimators are wide in the first two months because the number of incident cases are very low. For the last month or so when the diagnosed counts are relatively high, the 95% credible intervals are narrow.

### Application

**Fig 4.**
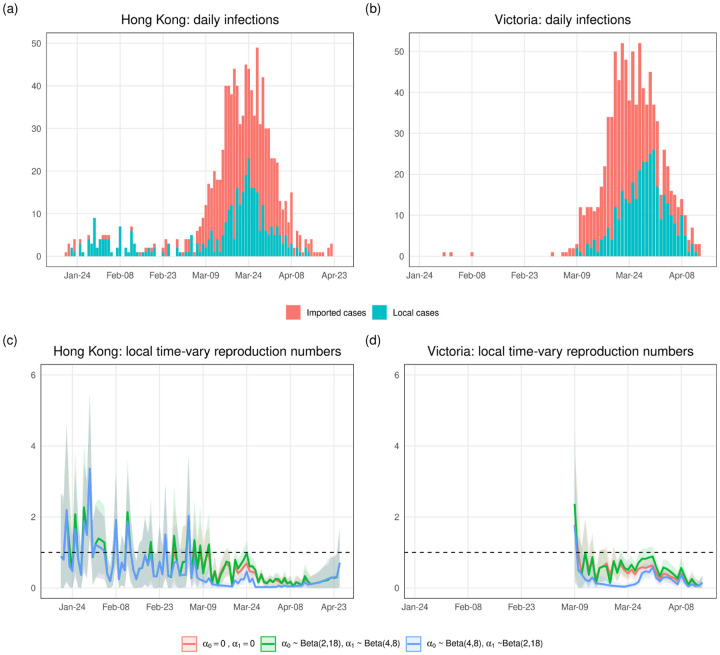
Epidemic curves of COVID-19 cases and estimations of local time-varying reproduction numbers in Hong Kong and Victoria. (a) The epidemic curve of daily cases of laboratory-confirmed SARS-CoV-2 infection in Hong Kong by symptom onset date and colored by case category. Asymptomatic cases are included here by date of confirmation. (b) The epidemic curve of the coronavirus disease cases in Victoria by sample collection date and colored by case category. (c) and (d) Estimations of local time-varying reproduction numbers under three scenarios: 1) no identification error, 2) *α*_0_ ~ Beta(2, 18) and *α*_1_ ~ Beta(4, 8) (around 10% imported cases are misclassified as local and around 33.3% local cases are misclassified as imported), 3) *α*_0_ ~ Beta(4, 8) and *α*_1_ ~ Beta(2, 18) (around 33.3% imported cases are misclassified as local and around 10% local cases are misclassified as imported). The bands are the 95% credible intervals.

We apply our proposed methods to surveillance data of COVID-19 in Hong Kong and Victoria. Fig 4 (a) and (b) show the daily local and imported cases counts in Hong Kong and Victoria. For Hong Kong data, [20] calculated the serial intervals using a gamma distribution and estimated shape and rate parameters of 2.23 and 0.37, respectively (corresponding to a mean of around 6 days and standard deviation of around 4 days). There is no specific serial interval that has been calculated for Victoria. Considering the epidemic curve in Victoria is relatively similar to that in Hong Kong, we use the same serial interval distribution when we estimate *R*^local^(*t*) in Victoria.

Fig 4 (c) and (d) show estimates for *R*^local^(*t*) under three scenarios: 1) no identification error, 2) small *α*_0_ and large *α*_1_, 3) small *α*_1_ and large *α*_0_. We run MCMC chains of 10,000 samples and the error bands are the 95% credible intervals. We can see that the estimated local time-varying reproduction numbers are quite different when the two identification error rates are about 10% and 30%. If we think we are more likely to misclassify local cases as imported, then we should trust the curve corresponding to scenario 2). If imported cases are more likely to be misidentified as local, then the curve corresponding to scenario 3) is reliable. And if we believe the identification error is close to zero, we should trust the estimate under scenario 1).

Ultimately, we see that the ability to account for identification error appropriately in reporting the local time-varying reproduction number can lead to substantially different conclusions than use of the original, noisy local time-varying reproduction number. These differences can then in turn be translated to decision making for public health response.

## Discussion

We have developed a general framework for estimation of the true local time-varying reproduction numbers in contexts wherein one has identified local and imported case counts with some error. Simulations demonstrate that substantial inferential accuracy by our estimators is possible when nontrivial error is present. And our application to epidemics in Hong Kong and Victoria shows that the gains offered by our approach over presenting the noisy local instantaneous reproduction number can be pronounced.

We have shown examples on a state/province level, but our method could be useful for cities, or more local settings, such as a university trying to determine if there is substantial local transmission occurring. Our approach requires daily numbers of local and imported cases, serial interval, and contact tracing data or other data to provide adequate information to estimate the misidentification rates.

We have pursued a Bayesian approach to the problem of estimating the local instantaneous reproduction number. The credible intervals are relatively wide when the number of cases is low. To improve the performance at low case incidence, Kalman filtering is a natural approach. Estimating the time-vary reproduction number by Kalman filtering is an emerging topic. For instance, [24] constructed a recursive Bayesian smoother for estimating the effective reproduction number from the incidence of an infectious disease in real time and retrospectively. However, one typically does not distinguish between local and imported cases in this setting.

The identification errors are informed by contact tracing survey data in our approach. If the data from the survey is categorical (e.g., we ask people where they were infected and attach some qualitative measure of our confidences that we think they are local cases), we can transform them into numerical values. For example, [25] proposed a method that converts categorical variables to numerical data for Gaussian distribution. We could modify the method to convert categorical variables to Beta distributed data. If the survey data is unavailable, using genomic data is a natural alternative. Genomic surveillance has been used to detect transmission clusters and to provide information on the possible source of individual cases [26–31].

We have showed the results of retrospective estimation. And it is computationally feasible to run MCMC on each day to obtain real time estimators; it takes about 5 minutes for the MCMC chain of 10,000 samples. To reduce the computational cost, one approach is adaptive MCMC methods [32, 33], which use the covariance structure of the posterior distribution to design proposal distributions. Other methods include stochastic Newton [34] and Riemannian manifold MCMC [35], which construct efficient proposals by local derivative information.
